# From Static Assessment to Dynamic Management: A Paradigm Shift in Biotoxicity Evaluation of Activated Sludge for Pharmaceutical Wastewater Treatment

**DOI:** 10.3390/toxics14050395

**Published:** 2026-05-04

**Authors:** Zhicheng Zhang, Wenyan Huang, Jinfeng Ding, Wenli Liu, Ruoxuan Xia, Worou Chabi Noel, Zhongjian Li, Hui Chen, Jun Yao

**Affiliations:** 1 Zhejiang Key Laboratory for Restoration of Damaged Coastal Ecosystems, Zhejiang International Science and Technology Cooperation Base for Biomass Resources Development and Utilization, School of Life Sciences, Taizhou University, Taizhou 318000, China; qiushizj@tzc.edu.cn (Z.Z.); 20241085701018@stu.tzc.edu.cn (W.H.); 13572955186@163.com (W.L.);; 2 Taizhou Environmental Science Design and Research Institute Co., Ltd., Taizhou 318000, China; dingjingfeng@zju.edu.cn; 3 Key Laboratory of Biomass Chemical Engineering of Ministry of Education, College of Chemical and Biological Engineering, Zhejiang University, Hangzhou 310027, China; zdlizj@zju.edu.cn; 4 Water Science and Technology Laboratory, National Water Institute, University of Abomey-Calavi, Abomey-Calavi 01 BP 526, Benin

**Keywords:** pharmaceutical wastewater, activated sludge, biotoxicity evaluation, knowledge-guided machine learning, resilience

## Abstract

The activated sludge process serves as the core barrier in pharmaceutical wastewater treatment, yet its stability is inherently challenged by the extreme complexity of influent composition and the unpredictability of toxic shocks, particularly under contract development and manufacturing organization (CDMO) operations. Current biotoxicity assessment methods face inherent trade-offs among timeliness, specificity, and matrix robustness, resulting in fragmented, reactive management that lacks predictive capacity. In response, this review critically synthesizes evidence on toxicity pathways and monitoring technologies, systematically evaluating their mechanistic basis and engineering applicability. Building on these findings, we propose a conceptual perception–cognition–response architecture that structures decision-making across three adaptive tiers: (i) a perception layer that tolerates false positives for rapid anomaly detection; (ii) a cognition layer that requires effect-based biological verification; and (iii) a response layer that authorizes resilience-oriented interventions. Rather than a linear pipeline, the three tiers form an adaptive feedback cycle that dynamically aligns monitoring intensity, verification depth, and response authority with real-time risk gradients and site-specific constraints. By explicitly linking biological mechanisms to assessment limitations and tiered decision rules, this review provides a hypothesis-generating roadmap that orients biotoxicity management from episodic, composition-based assessment toward adaptive, effect-driven control. The proposed framework is intended to guide future pilot validation, multi-sensor integration, and context-specific calibration, offering a unified narrative for advancing proactive biotoxicity control in complex pharmaceutical wastewater systems.

## 1. Introduction

**Figure 1:**
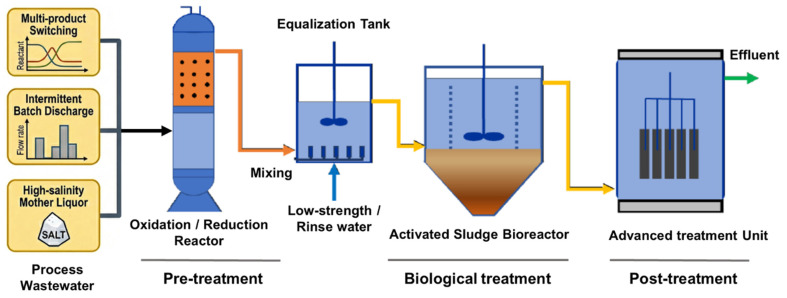
Schematic of a pharmaceutical WWTP under CDMO operation.

Pharmaceutical wastewater is widely recognized as one of the most challenging industrial effluents due to its complex composition, high salinity, and frequent fluctuations in both pollution load and toxicity [1]. Such volatility is exacerbated in the CDMO mode by rapid product switching, intermittent batch discharge, and high-salinity matrices [2]. Figure 1 depicts a typical treatment train: raw influent undergoes pretreatment (oxidation/reduction) before entering the activated sludge bioreactor—the core, yet most vulnerable barrier—where concentrated organic and saline shocks challenge microbial resilience, propagate nitrification failure, and accumulate sludge toxicity. This distributed vulnerability necessitates dynamic, effect-based biotoxicity evaluation beyond conventional static monitoring. Nevertheless, activated sludge remains the mainstream and cost-effective solution [3], despite its susceptibility to rapid process destabilization [4,5].

In conventional wastewater engineering practice, influent treatability and operational decisions are largely guided by bulk physicochemical indicators such as chemical oxygen demand (COD), biochemical oxygen demand (BOD_5_), ammonia nitrogen, and conductivity [6]. Although these indicators are useful for routine monitoring, they often fail to reflect the actual biological stress experienced by activated sludge. A typical mismatch arises when readily biodegradable organics coexist with potent toxicants (e.g., organic solvents, antibiotics, or reactive intermediates): the BOD_5_/COD ratio may suggest good biodegradability, yet microbial activity is severely inhibited [7]. Consequently, many pharmaceutical wastewater treatment plants (WWTPs) rely on conservative and energy-intensive pretreatment designed to reduce bulk chemical indicators, leaving the stability of downstream biological units uncertain.

To address this gap, a variety of biotoxicity assessment tools have been developed, ranging from standardized respirometry to rapid screening approaches [8]. More recently, molecular biology and omics-based diagnostics have been applied to elucidate microbial responses under toxic stress [9]. Despite these advances, current practice still follows a predominantly static, offline assessment paradigm involving discrete grab samples with lagging batch tests. Toxic shocks may occur within minutes, while conventional biological tests typically require hours to days, forcing operators into reactive “post-event diagnosis” rather than proactive prevention. In addition to timeliness limitations, two further barriers hinder effective toxicity management in activated sludge systems. First, online physicochemical sensor signals (e.g., pH, oxidation-reduction potential, dissolved oxygen, conductivity) are highly accessible, yet they are biologically non-specific. Fluctuations may be driven by load variation, operational disturbances, or sensor drift rather than genuine microbial inhibition, resulting in false alarms or missed events [10]. Second, even when toxicity is detected, actionable guidance is often lacking: most endpoints quantify inhibition (e.g., IC_50_) but do not directly translate into operational decisions [11,12]. As a result, WWTPs may become “data rich but information poor,” where abundant monitoring data does not reliably indicate what microorganisms are experiencing or what control actions could be taken.

These challenges indicate that existing toxicity evaluation systems are insufficient for the resilience demands of modern pharmaceutical wastewater treatment. In this paper, we provide a critical evidence synthesis restructuring biotoxicity knowledge into a decision-oriented narrative that links mechanism (what breaks), monitoring (what signals it), and action (what to do). Section 2 examines the mechanistic foundations of sludge toxicity. Section 3 evaluates current monitoring modalities across the timeliness–specificity–robustness trade-off space. Building on these findings, Section 4 synthesizes a tiered decision architecture comprising three adaptive tiers: rapid anomaly detection with tolerated false positives, effect-based biological verification coupled with risk forecasting, and resilience-informed intervention authorization with adaptive feedback. Section 5 outlines the framework’s conceptual implications as well as several interconnected frontiers. By anchoring each tier to documented evidence rather than fixed specifications, this review aims to provide a roadmap that orients biotoxicity management from reactive compliance testing to proactive, effect-driven resilience control.

## 2. Mechanisms of Biotoxicity to Activated Sludge Microorganisms

**Figure 2:**
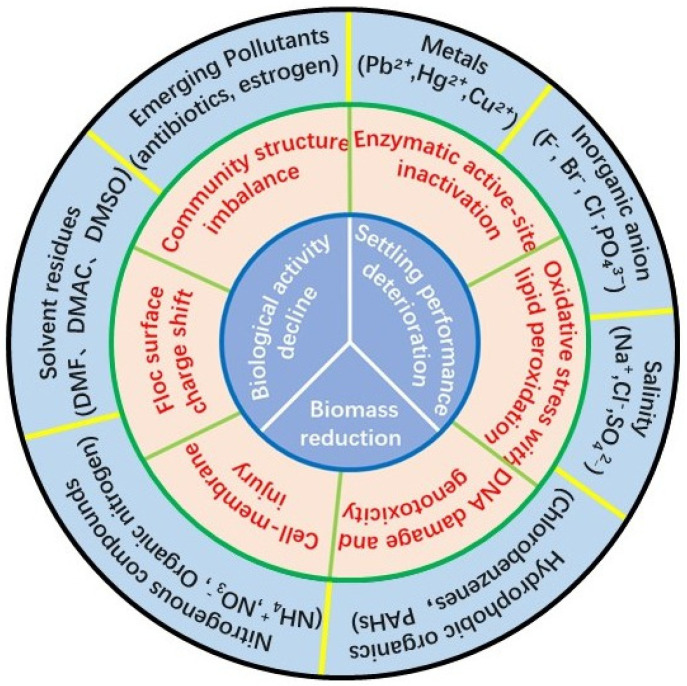
Conceptual framework linking chemical stressors in pharmaceutical wastewater (outer layer), hierarchical microbial response mechanisms in activated sludge (middle layer), and process-level performance deterioration (inner layer).

Activated sludge is a highly structured microbial ecosystem rather than a suspension of independent cells [13]. Microorganisms are embedded in flocs and protected by extracellular polymeric substances (EPS), which collectively form a multi-scale ecological barrier against environmental perturbations [14]. In pharmaceutical wastewater treatment, particularly under CDMO operation, the influent contains complex mixtures of organic solvents, antibiotics, intermediates, salts, and oxidative by-products. Table 1 summarizes representative chemical stressors frequently encountered in pharmaceutical wastewater, emphasizing those featured in the hierarchical framework (Figure 2, outer layer) while incorporating additional typical pharmaceuticals and intermediates. The hydrophobicity spectrum and speciation diversity illustrated here determine differential environmental fate, EPS partitioning, and cellular uptake, thereby directing the mechanistic pathways elaborated in Section 2.1, Section 2.2, Section 2.3, Section 2.4 and Section 2.5. Importantly, these mechanisms are interdependent [15]: early sub-lethal damage at the structural or cellular level may substantially reduce system resilience long before conventional functional endpoints (e.g., COD removal) deteriorate. Therefore, mechanistic understanding should be framed as a continuous chain linking chemical stressors, microbial responses, and process-level consequences, rather than isolated toxicant–endpoint relationships.

### 2.1. Disruption of the Ecological Barrier: EPS Degradation and Floc Destabilization

The EPS matrix serves as the fundamental ecological barrier, governing the structural stability and biosorption capacity of activated sludge [16]. In pharmaceutical wastewater treatment, particularly under the volatile CDMO mode, this barrier is subject to complex chemical stressors that trigger a mechanistic collapse of the microbial floc structure [17]. Specifically, chemical shocks from pharmaceutical intermediates and residual oxidants lead to the depletion and compositional shift of EPS, particularly targeting protein-like substances such as tyrosine and tryptophan-like components that act as molecular glue [18]. As these key components are degraded, the negative surface charge of microbial aggregates typically increases, elevating the electrostatic repulsion between cells. According to the extended Derjaguin Landau Verwey Overbeek (DLVO) theory, this increased energy barrier prevents effective microbial re-aggregation and promotes the disintegration of mature flocs into vulnerable pin-point structures [19].

This structural disintegration leads to the loss of the shielding–sorption equilibrium, which significantly exacerbates cellular exposure to toxicants. The EPS matrix normally functions as a sacrificial barrier, where its lipid and protein fractions provide hydrophobic regions to sequester potent pharmaceutical molecules. However, chemical stressors induce a rapid decline in the relative hydrophobicity (RH) of the sludge surface [19]. This shift, coupled with the collapse of the porous EPS framework, transforms the protective barrier into a porous bypass, exponentially increasing the effective diffusion coefficient of inhibitors toward the plasma membrane. Consequently, the compromised EPS-mediated cohesion not only triggers biomass washout but also facilitates direct toxicant–cell interactions, marking the transition from a resilient ecological system to a state of functional vulnerability [20].

### 2.2. Disruption of Cellular Integrity: Membrane Damage and Oxidative Stress

The cell membrane is the fundamental physical barrier maintaining microbial life; its integrity is a prerequisite for cell survival [21]. Toxicity via this pathway manifests as rapid, direct, and often irreversible damage, constituting a primary driver of acute microbial mortality. Toxic substances in wastewater bind to the cell membrane through electrostatic adsorption, lipid phase partitioning, or embedding, altering membrane fluidity and protein conformation, leading to structural destruction and functional loss. Hydrophobic organic compounds, especially organic solvents like N,N-dimethylformamide, acetonitrile, and phenol, can non-specifically partition into the phospholipid bilayer due to their lipophilicity [22]. This insertion increases membrane fluidity, disrupts its ordered structure, and leads to the loss of selective permeability. The consequence is the leakage of cellular contents (e.g., ions, ATP, nucleic acids) and the influx of external harmful substances, ultimately causing cell lysis [23]. Furthermore, residual surfactants can exert a more direct physical impact by solubilizing membrane lipids via emulsification, leading to severe structural disintegration [24].

Beyond physical disruption, oxidative stress amplifies membrane damage through radical-mediated lipid peroxidation. Reactive oxygen species may be introduced directly by oxidative wastewater constituents or generated in situ through metal-catalyzed reactions. Such oxidative aggression triggers lipid peroxidation chain reactions, causing irreversible damage to both the membrane lipid bilayer and membrane-bound proteins. Crucially, membrane disruption can act as an amplifier of mixture toxicity. The compromised permeability barrier facilitates the intracellular accumulation of other co-existing toxicants, transforming moderate exposure into severe metabolic inhibition [25].

### 2.3. Inhibition of Key Metabolic Pathways

Beyond physical damage, many toxicants disrupt central metabolic networks by intercepting electron flow, arresting enzymatic activity, and down-regulating functional genes across aerobic, nitrifying, and anaerobic guilds. The aerobic respiratory chain is the bioenergetic engine of activated sludge, and its disruption is a primary mode of action for many APIs and pretreatment residuals. Heavy metals and chlorophenols exert toxicity by binding to the sulfhydryl moieties of specific enzyme complexes [26,27]. These inhibitors block electron flux primarily at NADH dehydrogenase and cytochrome oxidase. This enzymatic blockade arrests the transport of protons across the membrane, thereby collapsing the proton motive force. The ultimate consequence is the decoupling of oxidative phosphorylation, manifesting as ATP synthesis arrest and a marked decrease in electron transport system activity [28].

Nitrifying micro-colonies are typically the most vulnerable functional group to chemical shocks. This vulnerability stems from site-specific inhibition of the two-step enzyme-catalyzed process. The first and rate-limiting step, ammonia oxidation, is intercepted by sulfur-containing organics, as well as specific API intermediates such as allylthiourea and aromatic amines [29]. These compounds act as competitive inhibitors or inactivators of the AMO enzyme. This biochemical inhibition is strongly correlated with a molecular response; namely, down-regulation of the *amoA* gene [30]. Similarly, the second step, nitrite oxidation, is targeted by stressors such as free nitrous acid. This induces *nxrA* gene down-regulation, thereby paralyzing NXR enzyme activity [31]. This concurrent inhibition results in rapid and often irreversible nitrification failure, leading to effluent ammonia and nitrite exceeding regulatory limits.

For treatment configurations utilizing anaerobic stages, methanogenic pathways represent another critical failure point. In the acetate pathway, which is often responsible for the majority of methane production, inhibitors such as long-chain fatty acids and certain sulfonamides target acetate activation. This halts the conversion of acetate to methane. Simultaneously, in the hydrogen pathway, chemical stressors like chloroforms disrupt the terminal step by inhibiting methyl-transferase activity [32]. These diverse inhibition points ultimately converge on the collapse of methane production, thereby diminishing both waste stabilization efficiency and energy recovery potential.

### 2.4. Genetic Damage and Ecological Selection Pressure

Beyond acute physiological toxicity, pollutants in pharmaceutical wastewater exert long-term, profound ecological and evolutionary pressures on microbial communities. Certain aromatic amines, nitroaromatics, and their degradation intermediates possess potent genotoxicity. They act as mutagens by intercalating into the DNA double helix, inducing strand breaks, or causing base-pair mismatches. Additionally, sub-inhibitory (or sub-lethal) concentrations of antibiotics, ubiquitous in pharmaceutical wastewater, serve as potent environmental selection factors. They eliminate sensitive strains while providing a significant competitive advantage to strains carrying corresponding Antibiotic Resistance Genes (ARGs), leading to the enrichment of the latter in activated sludge [33]. Crucially, the high microbial density and abundance of Mobile Genetic Elements (MGEs; e.g., plasmids, integrons) in activated sludge make it a “hotspot” for Horizontal Gene Transfer (HGT) of ARGs. This not only accelerates the emergence of multidrug-resistant bacteria but also turns treated effluent and excess sludge into significant sources of ARG dissemination into the environment [34].

### 2.5. Osmotic Stress

Pharmaceutical wastewater is often accompanied by high concentrations of inorganic salts (e.g., NaCl, Na_2_SO_4_), inducing osmotic stress as a broad-spectrum physiological shock. Microbial cells in hypertonic environments face severe risks of water loss and plasmolysis due to the significantly lower extracellular water potential [35]. To maintain cell turgor and normal physiological functions, microorganisms are forced to initiate complex osmoregulatory mechanisms, which consume substantial metabolic energy (ATP) to actively uptake or synthesize compatible solutes (e.g., betaine, proline) intracellularly. This forced diversion of energy significantly squeezes the budget originally allocated for cell growth and pollutant degradation, macroscopically manifesting as retarded growth rates and inhibited substrate degradation activity [36].

Consequently, early warning cannot rely on isolated chemical assays, motivating the tiered sensing architecture elaborated in Section 4.

## 3. Methods for Evaluating Biotoxicity of Pharmaceutical Wastewater Toward Activated Sludge

A wide spectrum of methods has been developed to assess the biotoxicity of wastewater toward activated sludge. These methods differ fundamentally in the biological level they interrogate, response timeliness, engineering robustness in harsh matrices, and data readiness for digital management systems. To align with the hierarchical mechanisms summarized in Section 2, this section reorganizes existing tools based on response time scale and engineering feasibility, which are the key determinants for dynamic toxicity management.

### 3.1. Conventional Physicochemical Screening Indicators

Conventional physicochemical indicators are the most widely used tools for preliminary wastewater treatability evaluation, routinely employed for influent screening, compliance reporting, and pretreatment design. Despite their operational practicality, these indicators quantify stoichiometric oxygen demand rather than microbial metabolic kinetics. Consequently, they cannot resolve synergistic or antagonistic toxicity in complex mixtures, nor capture sub-lethal physiological inhibition under realistic conditions [37]. While physicochemical indicators are indispensable for routine screening, they are insufficient as stand-alone metrics for toxicity risk management in highly dynamic pharmaceutical wastewater systems.

### 3.2. Real-Time Online Monitoring and Soft-Sensing (Seconds to Minutes)

#### 3.2.1. Surrogate Physicochemical Sensor Arrays

Industrial-grade sensors for pH, dissolved oxygen (DO), oxidation–reduction potential (ORP), conductivity, and temperature constitute the most robust monitoring layer in industrial WWTPs. Although these sensors do not directly measure toxicity, they capture system-wide metabolic and physicochemical shifts induced by microbial activity and influent perturbations [38]. For example, abrupt changes in DO dynamics (e.g., slope changes, delayed recovery after aeration control) may indicate suppressed respiration, while ORP and pH fluctuations may reflect altered redox states and acid–base buffering capacity. Conductivity provides a direct proxy for osmotic stress risk and often correlates with salinity shocks in pharmaceutical wastewater.

However, surrogate sensors suffer from low biological specificity. Similar signal patterns may be caused by non-toxic load variations, operational disturbances, or sensor drift, leading to false alarms. Consequently, their optimal role is not toxicity quantification but continuous surveillance and anomaly detection, providing high-frequency time-series data suitable for machine learning models.

#### 3.2.2. Pattern Recognition and Anomaly Detection for Early Warning

Given the non-specificity of surrogate sensors, data-driven algorithms are increasingly applied to extract toxicity-related “fingerprints” from multi-parameter time series. These approaches move beyond absolute sensor values, instead quantifying deviations in temporal dynamics, cross-correlation structures, and process inertia. Techniques range from multivariate statistical process control (MSPC) and dynamic principal component analysis (PCA), which detect departures from normal operational covariance structures, to deep learning architectures such as variational autoencoders (VAE) and long short-term memory (LSTM) networks that capture non-linear, long-range temporal dependencies in sensor trajectories [39,40]. The key value of anomaly detection lies in enabling early warning before effluent deterioration occurs. Nonetheless, model generalization remains challenging due to site-specific influent patterns and the scarcity of labeled toxicity events.

### 3.3. Rapid Bioassays and Online Biosensors (Minutes to Hours)

#### 3.3.1. Respirometry-Based Inhibition Monitoring

Respiration inhibition tests, standardized as ISO 8192:2007, quantify wastewater biotoxicity by measuring the reduction in oxygen uptake rate (OUR) or specific oxygen uptake rate (SOUR) of activated sludge after exposure [41]. As respiration integrates the overall metabolic activity of aerobic heterotrophs and, depending on the protocol, nitrifiers, respirometry is widely regarded as a benchmark effect-based indicator for assessing microbial inhibition [42,43]. WTW respirometers were employed to capture oxygen consumption curves, based on which approaches were proposed to quantify inhibition intensity by comparing endogenous respiration baselines with post-exposure respiration profiles, enabling differentiation between inhibition of carbon oxidation and nitrification. The applicability of respirometry to pharmaceutical wastewater has been demonstrated in early studies [4], while recent works have improved reproducibility through optimized operational parameters [44]. ISO 8192 is technically an at-line assay amenable to automation, yet its practical deployment remains confined to controlled laboratory conditions. Interpretation may be confounded by substrate limitation, temperature variation, and biomass heterogeneity; measurement stability is further challenged by high salinity, foaming, and matrix effects commonly encountered in pharmaceutical wastewater.

#### 3.3.2. Nitrification Inhibition Assays and Nitrifier-Based Detectors

Nitrification inhibition assays, standardized as ISO 9509:2006, evaluate the impact of toxicants on ammonia-oxidizing and nitrite-oxidizing bacteria by monitoring ammonia/nitrite conversion rates or the associated oxygen consumption. Because nitrifiers are among the most sensitive functional guilds in activated sludge, nitrification inhibition tests often provide earlier warning of toxicity than bulk respiration indicators, rendering them particularly relevant for pharmaceutical wastewater where nitrification failure is frequently the first observable symptom of biotoxic shocks. To enhance engineering applicability, nitrifier-based detection has been explored in immobilized or biosensor formats [45]. For example, an automated biodetector (ABTOW) has been reported for continuous toxicity monitoring, which immobilizes nitrifying bacteria on open-pore polyurethane foam and uses changes in oxygen consumption as a proxy for nitrification activity in response to xenobiotics [46]. Overall, nitrification inhibition methods are relatively simple, rapid, and sensitive, and they can exhibit strong responses to specific inhibitors such as chromium and mercuric chloride (HgCl_2_). Nevertheless, these assays remain confined to the single dimension of nitrogen transformation and may overlook broader ecological impacts on other key functional groups, including heterotrophs, floc-forming bacteria, EPS-producing populations, and polyphosphate accumulating organisms (PAOs) [47].

#### 3.3.3. Luminescent Bacteria Toxicity Assays (LBTA)

Luminescent bacteria assays quantify acute toxicity by measuring the inhibition of bioluminescence in model strains, specifically *Aliivibrio fischeri* (formerly *Vibrio fischeri*). This method, standardized as ISO 11348-3, offers rapid results (typically within 15–30 min), enabling near-real-time screening for broad-spectrum acute toxicity. LBTA is useful for detecting broad-spectrum acute toxicity and is often applied for influent screening or emergency response. Research demonstrates that *A. fischeri* exhibits a robust dose–effect response often following a sigmoid S-curve to various pharmaceutical residues, particularly antibiotics (e.g., ampicillin, ciprofloxacin) and their reactive intermediates [48].

However, LBTA application to pharmaceutical wastewater faces several methodological constraints. First, the ecological relevance to activated sludge is limited; as *A. fischeri* is a marine organism, the requirement for sample salinity adjustment (typically to 2% NaCl) alters the physicochemical matrix, potentially affecting toxicant bioavailability and deviating from the freshwater environment of activated sludge [49]. Second, optical interference remains a critical bottleneck; the high turbidity and chromaticity characteristic of pharmaceutical effluents can absorb or scatter bioluminescence, leading to false positives unless rigorous color-correction protocols are employed. In addition, false negatives may occur due to hormesis (low-dose stimulation), a phenomenon frequently observed where sub-lethal concentrations of antibiotics stimulate rather than inhibit bacterial luminescence [50]. Furthermore, comparative analyses demonstrate that Vibrio fischeri-based assays may yield toxicity rankings diametrically opposed to activated sludge inhibition results for specific surfactants, rendering them unreliable as sole predictors of biological treatment impacts [42,51]. Finally, differential sensitivity remains a concern, as certain toxicants potent against autotrophic nitrifiers may elicit only negligible responses in these heterotrophic luminescent bacteria.

#### 3.3.4. Microbial Electrochemical Sensors

Microbial electrochemical sensors, particularly those based on Microbial Fuel Cells (MFCs), leverage the principles of extracellular electron transfer (EET) to transduce microbial metabolic activity into real-time digital electrical signals (e.g., current or voltage). The fundamental detection mechanism relies on the fact that toxicants inhibit the metabolic activity of electroactive biofilms, precipitating a measurable decay in current output. Due to their capability to generate continuous, high-frequency time-series data, these sensors are uniquely poised for integration with artificial intelligence (AI) and digital twin frameworks [52].

Despite their conceptual elegance and potential for online deployment, widespread application is currently hindered by limited sensitivity and susceptibility to signal interference. A critical bottleneck is the “masking effect” caused by organic load fluctuations: the presence of readily biodegradable organics (e.g., acetate, glucose) can stimulate metabolic current generation, effectively counteracting and obscuring the inhibitory signals induced by co-existing toxicants. This creates a high risk of false negatives in complex matrix monitoring [53]. To mitigate these limitations, recent research has focused on enhancing signal-to-noise ratios through advanced anode modification (e.g., nanomaterials) and innovative reactor designs. The development of miniature, low-cost Single-Chamber MFCs (SCMFCs) and paper-based devices has enabled the successful detection of heavy metals like cadmium and lead [54]. Nevertheless, challenges regarding the long-term stability of bio-electrodes and the decoupling of toxicity signals from organic load variations remain active frontiers of research.

#### 3.3.5. Targeted Biosensors and Molecular Probes

Biosensors tailored for specific pollutant detection typically comprise two core functional modules: a biorecognition element (bioreceptor) and a physicochemical transducer. The recognition element may consist of biological macromolecules (e.g., enzymes, antibodies, DNA/RNA), whole cells, or synthetic receptors such as aptamers and molecularly imprinted polymers [55,56]. The transducer converts the specific binding event between the receptor and the target analyte into a measurable signal (optical, electrochemical, or piezoelectric). For instance, fluorescence resonance energy transfer (FRET)-based biosensors utilize fluorophore-labeled aptamers that undergo conformational changes upon binding to specific toxicants, generating a quantifiable modulation in fluorescence intensity [57]. These targeted molecular probes enable high specificity and potential in situ monitoring, yet their engineering application is severely constrained by matrix interference and biofouling in pharmaceutical wastewater [58]. The complex matrix rapidly deactivates biological receptors or passivates sensor surfaces, limiting operational lifespan. This specificity–robustness trade-off exemplifies a broader limitation of current early warning tools: they signal anomaly presence without revealing causative factors or consequences for activated sludge stability. Operators respond to alarms without adequate information to guide regulatory interventions.

### 3.4. Mechanistic and Molecular Diagnostics (Hours to Days)

#### 3.4.1. Cell Integrity and Oxidative Stress Assays

Live/dead staining combined with flow cytometry quantifies membrane integrity loss, providing direct evidence of acute cellular damage [59]. Additional assays targeting reactive oxygen species or lipid peroxidation biomarkers help diagnose oxidative stress mechanisms contributing to toxicity. These methods are particularly useful for linking toxicity events to cellular damage pathways, as ROS production and lipid peroxidation are common responses to toxicants. For example, a method was developed to assess whole-water samples by directly exposing fish cells, such as the rainbow trout gill cell line (RTgill-W1), to untreated water [60]. This approach revealed cytotoxicity in certain effluents while others showed no such effects. Mitochondrial toxicity was assessed using the AREc32 cell line, where disruption of mitochondrial membrane potential (MMP) in WWTP effluent samples suggested oxidative stress effects [61]. These assays offer promising tools for assessing water quality in a biologically relevant context. However, these methods require sample pretreatment, including floc dispersion, and are sensitive to matrix interference, such as particulates or dissolved organics in wastewater.

#### 3.4.2. qPCR and Functional Gene Monitoring

Quantitative PCR enables tracking of functional guilds and stress-related genes, such as *amoA* for nitrifiers and antibiotic resistance genes (ARGs) for resistance selection. Gene-level endpoints provide strong ecological relevance and allow differentiation of targeted inhibition from general metabolic stress. For example, qPCR-based monitoring has revealed that antibiotic exposure can induce transient upregulation of *amoA* transcription in ammonia-oxidizing bacteria, which reflects an adaptive enzymatic response during metabolic degradation of micropollutants rather than irreversible functional collapse [62]. Similarly, ARG- and mobile genetic element (MGE)-targeted qPCR has been widely applied to evaluate antimicrobial resistance (AMR) risks in wastewater systems [63].

However, conventional DNA-based qPCR reflects total genetic abundance without discriminating viable from dead hosts, potentially obscuring actual resistance behavior. Recent advances combining propidium monoazide treatment with qPCR have demonstrated that a substantial fraction of intracellular ARGs and MGEs can be associated with dead biomass, while viable bacteria and mobile elements jointly drive ARG enrichment [64]. This refinement enables attribution of AMR risks to active microbial populations. Coordinated shifts in functional genes (e.g., *nosZ*, *napAB*) and ARG subtypes under compound stresses highlight that resistance selection often co-occurs with nitrogen transformation pathways, emphasizing the integrative value of gene-level surveillance [63]. Nevertheless, qPCR requires DNA/RNA extraction, careful normalization, and laboratory-based workflows. Its temporal resolution and operational complexity limit its applicability for real-time control, positioning qPCR primarily as a tool for periodic surveillance, mechanistic diagnosis, and forensic confirmation rather than continuous process regulation.

#### 3.4.3. Omics Technologies

Omics approaches offer the most comprehensive view of microbial community structure and functional response under complex environmental stressors. Metagenomics elucidates shifts in community composition, functional potential, and resistance reservoirs in response to toxic or non-biodegradable pollutants, while transcriptomics captures rapid regulatory responses at the gene expression level. Proteomics further bridges genotype-phenotype gaps by linking stress exposure to enzymatic activity and pathway-level regulation. Together, these multi-omics tools enable systematic characterization of microbial consortia, revealing functional redundancy, metabolic specialization, and stress adaptation mechanisms that are inaccessible through single-gene assays [65,66]. Cross-validation across omics layers provides robust evidence linking microbial regulation to macroscopic outcomes such as methane yield, contaminant removal, or stress resilience. Omics further facilitate biomarker discovery by identifying key taxa, genes, and proteins consistently responding to environmental perturbations, supporting hypothesis-driven monitoring and the mechanistic grounding required for interpretable risk-forecasting models [67].

Despite their explanatory power, omics technologies remain constrained by high cost, analytical complexity, and long data-processing cycles. Consequently, they are best positioned as research-intensive diagnostic tools for mechanism elucidation, model calibration, and intervention assessment—functions that inform rather than replace real-time operational control.

### 3.5. Predictive and Computational Approaches

#### 3.5.1. QSAR-Based In Silico Toxicity and Risk Screening

Quantitative structure-activity relationship (QSAR) models predict toxicity- or performance-related endpoints based on molecular descriptors such as hydrophobicity (e.g., log *K*ow), polarity, electronic distribution, and molecular topology [68]. QSAR approaches are particularly valuable in contexts where experimental ecotoxicological data are scarce, such as for APIs, their transformation products, or other contaminants of emerging concern (CECs). By leveraging existing physicochemical and toxicological databases, QSAR enables rapid in silico screening and prioritization of potentially hazardous compounds, thereby supporting regulatory risk assessment and reducing reliance on time-consuming and resource-intensive bioassays [69]. In wastewater and engineered biological systems, QSAR has been applied to estimate toxicity thresholds for activated sludge processes, providing a pragmatic means to screen influent toxicity and anticipate biological process upsets [70]. QSAR-assisted mixture toxicity assessments further identify substances of concern in complex effluents when experimental data for many components are unavailable [71].

Nevertheless, QSAR performance depends strongly on the quality and representativeness of training datasets and is inherently constrained by its reliance on additive or single-compound assumptions. The presence of unknown constituents, transformation products, and synergistic effects in complex mixtures—typical of CDMO wastewater—can lead to underestimation of actual toxicity. Accordingly, QSAR is best suited as a first-tier screening and prioritization tool, complementing rather than replacing experimental toxicity testing and effect-based monitoring.

#### 3.5.2. Data-Driven Prediction for Dynamic Risk Forecasting

Data-driven approaches, particularly machine learning (ML) models, have gained prominence for predicting biotoxicity risks in wastewater treatment systems, facilitating proactive forecasting of process inhibition and effluent quality deterioration. These methods integrate heterogeneous data, including online sensor streams, physicochemical parameters, and biological endpoints, to model complex influent–toxicity relationships. For tabular datasets from discrete assays, ensemble methods such as random forests (RF) and support vector machines (SVM) classify toxicity levels or predict inhibition thresholds [72,73]. Boosting algorithms, notably extreme gradient boosting (XGBoost) and adaptive boosting (AdaBoost), excel in managing imbalanced data and non-linear interactions [74]. An XGBoost-based framework predicted integrated toxicity units from 14 parameters across 99 samples from 21 industries, achieving R^2^ > 0.94 and RMSE of 3.5 TU, with conductivity, pH, and heavy metals as key features [75]. SHAP analysis further revealed feature influences, demonstrating interpretable ML’s potential for microbial mixture effects in activated sludge [76].

Comprehensive reviews underscore ML’s versatility across endpoints from acute toxicity to ecotoxicity, advocating QSAR-ML hybrids for high-throughput screening [77]. Tools such as MS2Tox predict ecotoxicity from mass spectra of unidentified contaminants, while hybrid CNN-LSTM models enhance predictions of wastewater parameters like DO and TDS [78,79]. Nevertheless, deployment in dynamic WWTPs encounters challenges: labeled toxicity event scarcity (<10% of datasets), class imbalance, limited interpretability, and generalization issues under covariate shifts. Mitigation strategies include feature selection, data augmentation, transfer learning from data-rich allied sectors (e.g., petrochemical or municipal WWTPs), and explainable AI (e.g., SHAP) for mechanistic insights [80]. However, integrating knowledge, such as activated sludge kinetics and hierarchical mechanisms (Section 2), remains nascent, necessitating hybrid frameworks elaborated in Section 4.

### 3.6. Comprehensive Comparison of Existing Methods

As illustrated in Table 2, current biotoxicity evaluation methods for activated sludge have evolved into a diversified technological landscape, ranging from molecular to population levels and from offline snapshots to online monitoring. Yet, no single method satisfies the demands of dynamic process control in modern pharmaceutical wastewater treatment.

**Figure 3:**
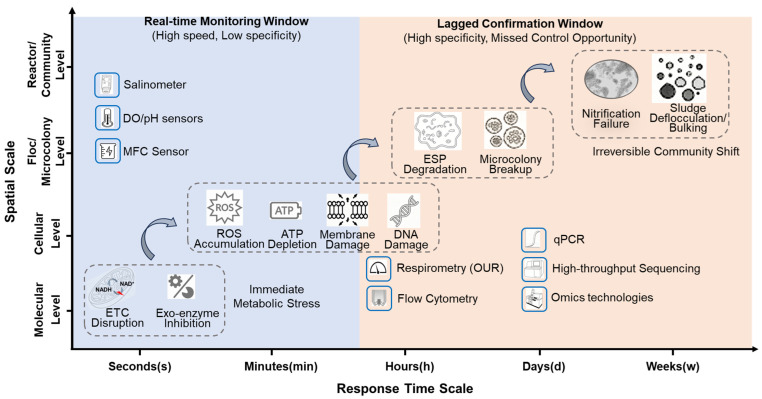
Cross-scale and time-dependent toxicity responses in activated sludge systems, highlighting the inherent limitations of static assessment. Curved arrows indicate the temporal progression of damage, reflecting the sequential order of toxicity manifestation from immediate metabolic stress to irreversible community shifts. *Abbreviations*: ETC, electron transport chain; ROS, reactive oxygen species; OUR, oxygen uptake rate; qPCR, quantitative polymerase chain reaction; ATP, adenosine triphosphate; MFC, microbial fuel cell; NGS, next generation sequencing.

The core trade-off exists between response timeliness and ecological relevance: molecular and cellular assays (qPCR, flow cytometry, proteomics, omics) deliver high mechanistic resolution and strong ecological relevance to native sludge communities, yet require hours to days, rendering them unsuitable for real-time control; by contrast, physicochemical sensor arrays provide second-level continuous data but lack biological specificity, frequently generating false alarms driven by non-toxic load variations. A second critical limitation is data readiness for digital twins and machine learning, where high-frequency, structured time-series signals from physicochemical sensors and MFC-based biosensors integrate seamlessly into AI workflows, whereas most bioassays produce discrete, low-frequency, or unstructured outputs (e.g., batch respirometry curves or image-based viability data) that demand extensive preprocessing and hinder continuous modeling. A third persistent conflict lies between analytical sensitivity and engineering robustness, as laboratory-optimized biological probes often suffer rapid biofouling, electrode passivation, and matrix interference in high-salinity, high-organic-load pharmaceutical effluents, leading to unacceptable maintenance burdens and signal instability. As illustrated in Figure 3, these methods map unevenly across scales: instantaneous molecular stress responses are captured by rapid but non-specific sensors, while long-term community succession and genetic selection are only accessible through lagging, high-resolution diagnostics, with no single category spanning the full continuum with adequate timeliness, specificity, and robustness.

To address this gap, transitioning from reactive compliance testing to proactive resilience control requires a hierarchical decision logic that strategically integrates continuous effect-proximal sensing with targeted mechanistic verification.

## 4. A Conceptual Architecture for Biotoxicity Management

Building on the mechanistic insights (Section 2) and methodological trade-offs (Section 3), this section synthesizes existing knowledge into a conceptual decision framework intended to prioritize research directions for dynamic biotoxicity management. We propose a perception–cognition–response architecture comprising three sequential decision tiers: (i) rapid anomaly detection with tolerated false positives; (ii) effect-based confirmation coupled with risk forecasting; and (iii) resilience-informed intervention authorization. All described components—including sensor configurations, algorithmic approaches, threshold logic, and intervention protocols—represent hypothesized technical solutions derived from the current literature, awaiting systematic pilot validation.

### 4.1. Tier 1: Perception Layer for Rapid Anomaly Detection

The perception layer serves as the first decision tier, performing continuous surveillance and anomaly pattern recognition. Conceptually, this tier could transform raw sensor streams into a Virtual Toxicity Index (VTI) through adaptive signal processing. The VTI would estimate a minimum set of current operational states necessary for triggering escalation, including instantaneous performance deviation, detected stress intensity, and observed resilience indicators—all derived from high-frequency observables including DO slope changes, ORP fluctuation amplitude, conductivity variation rates, pH deviation magnitude, or other sensor signals. This index construction aligns with the SIMONI strategy’s principle of integrating heterogeneous observables into effect-proximal trigger values for tiered decision-making [89,90], and draws upon multi-sensor consensus frameworks that dynamically weight sensor contributions based on real-time information gain [91,92]. The primary function is rapid anomaly flagging with tolerated false positives, explicitly without trajectory extrapolation.

However, translating these raw signals into biologically meaningful alerts may encounter a fundamental observability challenge: distinguishing instrument artifacts from genuine toxicity-induced signals. In pharmaceutical effluents, high salinity induces baseline drift and matrix interference, while high suspended solids and foam accelerate biofouling and electrode passivation [93,94]. Adaptive filtering techniques have demonstrated potential for dynamic correction in saline matrices [91], though their transferability to CDMO toxicity monitoring awaits pilot validation. Multi-sensor consensus logic could reduce artifact-driven false alarms, but the specific fusion algorithms, threshold calibration, and fail-safe protocols to prevent misinterpreting equipment failure as biological stress all require extensive site-specific investigation [95]. Empirical evidence from full-scale systems further indicates that predictive models degrade significantly under real-world concentration variability, reinforcing the need for dynamic baseline recalibration in any deployed virtual index [96]. Accordingly, the VTI risk stratification into graduated response zones, as well as the mathematical formulations of the state variables, remain active research questions pending operational calibration.

Upon VTI persistence above locally calibrated thresholds for a sustained observation window, Tier 1 would output a timestamped anomaly flag and pre-processed sensor data for Tier 2 analysis. The specific duration of persistence validation requires calibration against historical databases to optimize the trade-off between detection latency and false positive suppression.

### 4.2. Tier 2: Cognition Layer for Effect-Based Verification and Risk Forecasting

The cognition layer serves as the second decision tier, receiving Tier 1 anomaly alerts and pre-processed sensor streams. Its primary function is to transform these heterogeneous inputs into actionable operational intelligence through two sequential operations: effect-based biological confirmation and predictive risk forecasting. Conceptually, this tier could integrate rapid bioassay verification (e.g., respirometry inhibition or nitrification inhibition assays from Section 3.3) with machine learning-driven trajectory prediction, translating discrete biological endpoints into risk profiles with uncertainty quantification.

However, realizing this integration may also face two fundamental research challenges. First, the scarcity of labeled toxicity events for model training: biotoxicity incidents in CDMO operations are statistically rare, creating severe class imbalance that undermines supervised learning performance. Semi-supervised learning and synthetic augmentation techniques show promise for model development with limited labeled data [97,98], though their efficacy for pharmaceutical wastewater matrices awaits rigorous validation. Second, even when data are available, the trust deficit associated with purely data-driven predictions: without mechanistic grounding, black-box forecasts risk generating physiologically implausible trajectories. Recent advances in environmental chemistry demonstrate that integrating Shapley Additive Explanations with quantum chemical descriptors can align model interpretability with established electron-transfer theory, offering a methodological reference for embedding mechanistic constraints into predictive analytics [99]. Physics-informed neural networks are emerging to embed activated sludge kinetic constraints and uncertainty quantification into predictive analytics [100], yet their adaptation to dynamic CDMO forecasting—with rapid product switching and non-stationary influent patterns—remains nascent. As a complementary lightweight approach, minimal-state predictive modeling could further support scenario exploration by estimating essential decision variables within fundamental observability constraints [101,102,103], though the specific variable selection and state-space reduction rules require calibration against Tier 2 biological endpoints. Pending resolution of these challenges, the optimal fusion architecture between biological verification and predictive modeling remains an active research question. Threshold criteria distinguishing high- from low-confidence forecasts require operational calibration for each CDMO context.

Upon achieving sufficient predictive confidence and biological verification, Tier 2 would output toxicity trajectories with uncertainty bounds to Tier 3. High-uncertainty predictions would warrant extended observation protocols—prolonged monitoring duration, additional bioassay replication, or cross-validation against historical analogs. Low-uncertainty forecasts might enable proactive preparedness (pre-positioning of buffering capacity or alternative influent routing), though specific escalation criteria require operational validation. If biological verification negates the Tier 1 anomaly, the system would flag a false positive and feedback threshold adjustment recommendations to Tier 1. If Tier 2 confirmation is unavailable (e.g., due to equipment failure, reagent depletion, or matrix interference exceeding assay limits), the system falls back to Tier 1 protocols, with predictive modeling suspended until verification is restored. Mandatory effect-based confirmation provides essential evidentiary grounding before Tier 3 activation.

### 4.3. Tier 3: Response Layer for Intervention Authorization and Adaptive Learning

The response layer serves as the final decision tier, receiving Tier 2 verified trajectories and uncertainty bounds. As a hypothesized operational logic, Tier 3 would conceptually require sustained inhibition beyond functionally critical thresholds before authorizing actuation [104]. Its primary function is to translate verified risk intelligence into resilience-oriented operational responses. This prioritizes microbial functional integrity over rapid effluent compliance. The underlying logic is that effluent compliance without functional resilience risks cascading failure. Specific modulation strategies, dosing logic, and sequencing rules remain contingent on local plant configuration. All await pilot-scale validation.

However, translating predictive intelligence into timely action while avoiding hazardous trial-and-error may face a fundamental deployment tension [105]. Systematic outcome evaluation could close the virtual–physical gap. The feedback-driven approach represents a promising solution [106], yet such protocols remain largely hypothetical for full-scale systems. High-resolution mechanistic diagnostics (e.g., effect-directed analysis, molecular resistance tracking) could elucidate causative pathways [107,108]. However, their integration into real-time algorithms awaits extensive field demonstration. Pending such validation, the feedback architecture linking intervention outcomes to model refinement, as well as safeguards against destabilizing feedback cycles, remain active research questions.

Upon successful intervention execution, Tier 3 would systematically feed outcomes back to Tier 2 for model refinement. Conceptually, false positives would inform specificity adjustments, while confirmed events could enrich training datasets, though technical protocols for continuous learning loops remain to be established. If intervention outcomes contradict predictive trajectories, the system would flag model degradation and suspend adaptive updates pending Tier 2 re-verification. If authorization is declined, the system returns to Tier 2 extended observation protocols. The three tiers are thus conceptualized to operate as a conditional cycle: Tier 1 triggers surveillance escalation, Tier 2 verifies and forecasts, Tier 3 authorizes and learns, thus feeding refined thresholds back to Tier 1 and enriched datasets back to Tier 2.

## 5. Conclusions

This review critically synthesizes the multi-scale mechanisms of biotoxicity in pharmaceutical wastewater and the inherent trade-offs of current assessment methodologies. The results consistently demonstrate that no single monitoring modality can simultaneously deliver the timeliness required for early detection, the specificity demanded for mechanistic verification, and the robustness needed for continuous field deployment. To address this methodological gap, we integrate these findings into a conceptual perception–cognition–response architecture. Rather than prescribing fixed operational parameters, this framework establishes a hierarchical decision logic that aligns signal acquisition, biological confirmation, and intervention authority with real-time risk gradients. By explicitly linking rapid cellular stress to delayed functional impairment, the proposed structure offers a coherent pathway to shift biotoxicity management from episodic, composition-based testing toward continuous, effect-driven resilience control.

It is important to clarify that the architectural components and decision rules presented herein are hypothesis-generating concepts derived from current research, not validated engineering standards. Their practical implementation requires systematic pilot-scale validation, site-specific calibration to local influent matrices and plant configurations, and continuous integration of operational feedback. We therefore direct future efforts toward several interconnected frontiers: (i) the standardization of effect-proximal sensing streams and adaptive threshold protocols for multi-tier decision gates; (ii) the development of interpretable risk-forecasting models that embed activated sludge kinetics and uncertainty quantification; and (iii) the creation of open benchmark datasets spanning diverse CDMO operational scenarios to enable cross-site model validation and transfer learning. Realizing these advances will require transdisciplinary collaboration bridging environmental engineering, data science, microbial ecology, and control theory disciplines to transform biotoxicity management from reactive compliance testing to proactive, effect-driven resilience control.

## Figures and Tables

**Table 1:** Representative chemical stressors in pharmaceutical wastewater.

Compound	Abbreviation	Molecular Formula	Application	Log Kow	pKa
Ciprofloxacin	CIP	C_17_H_18_FN_3_O_3_	Fluoroquinolone antibiotic	−1.10	6.09 (carboxyl); 8.74 (piperazinyl)
Erythromycin	ERY	C_37_H_67_NO_13_	Macrolide antibiotic	3.06	8.8
N,N-Dimethylformamide	DMF	C_3_H_7_NO	Organic solvent	−0.93	nonelectrolyte
N,N-dimethylacetamide	DMAC	C_4_H_9_NO	Organic solvent	−0.77	nonelectrolyte
Dimethyl sulfoxide	DMSO	C_2_H_6_OS	Organic solvent; cryoprotectant	−1.35	35 (C-H acidity)/ −1.5 (conjugate acid)
Acetonitrile	ACN	C_2_H_3_N	Organic solvent	−0.34	25 (C-H acidity)/ −10.1 (conjugate acid)
4-aminophenol	4-AP	C_6_H_7_NO	Synthetic intermediate	0.04	5.29 (phenolic hydroxyl)/10.30 (amino group)
Polycyclic aromatic hydrocarbons	PAHs	C_10_H_8_–C_22_H_12_	Organic solvent	3.30~7.01	nonelectrolyte
Ibuprofen	IBU	C_13_H_18_O_2_	NSAID	3.97	4.91
Tetracycline	TC	C_22_H_24_N_2_O_8_	Tetracycline antibiotic	−0.19	7.68
Sulfamethoxazole	SMX	C_10_H_11_N_3_O_3_S	Sulfonamide antibiotic	0.89	1.85 (amino)/5.60 (sulfonamide group)

**Table 2:** Overview of biotoxicity methods for activated sludge.

**Methods**	Mechanisms	Response Timeline	Reliability	Cost	Online Capability	Ecological Relevance	Data Readiness for Digital Twins	Major Limitation	Applications Niche	References
Surrogate physicochemical sensor arrays	Reflects bulk physicochemical fingerprints	Real-time	Low	Low	Online	Low to Medium	Very High (Structured time-series data; seamless model integration)	No direct biological meaning	Pre-screen, auxiliary data	[38,81]
Respiration inhibition (ISO 8192)	Aerobic respiration rate: Indicates overall metabolic activity of aerobic sludge	0.5–2 h	High	Medium	At-line (automatable)	High (Directly measures core activated sludge function)	Medium (Requires dedicated analyzer; susceptible to drift)	Aerobic heterotrophs and nitrifiers only	Regulatory benchmark, plant monitoring	[41,44]
Nitrification inhibition (ISO 9509)	Ammonia/Nitrite oxidation rate: Indicates specific enzyme activity	2–4 h	High	Medium	At-line (automatable)	Very High (Targets the most sensitive functional guilds)	Low to Medium (Often requires manual sampling or complex wet-chem auto-analyzers)	Nitrifying guilds only	NH_3_-oxidation risk assessment	[82,83]
LBTA	Luciferase activity: Indicates general metabolic state	10–30 min	Medium	Low	Offline/ At-line	Low (Uses specific strains, not representative of native sludge community)	Low (Batch data points; difficult to integrate into continuous flow)	Color/turbidity interference	Quick screen, field alert	[50,84]
MFC-biosensor	Extracellular electron transfer: Indicates bio-electrochemical activity	5–30 min	Medium	Low–medium	Online/ At-line	Medium to High (Can inoculate with native plant sludge)	High (Voltage/current signals are direct digital inputs for AI)	Low sensitivity, drift	On-line trend indication	[53,85]
Live/Dead staining flow cytometry	Membrane integrity: Indicates physical damage	1–3 h	Medium	High	Offline	High (Visualizes cell viability status)	Low to Medium (Image/scatter plot data requires complex preprocessing)	Complex pretreatment (floc disintegration); High equipment cost	Quantitative viability and membrane integrity assessment	[59,60]
qPCR and high-throughput sequencing	Functional gene abundance/expression	qPCR: 4–6 h; HTS: 1–3 d	High	High	Offline	Very High (Specific identification of functional gene response)	Very Low (Complex, low-frequency data; suitable for calibration, not control)	RNA/DNA extraction needed	Functional genes, ARG tracking	[63,86]
Omics technologies	Metagenomics/transcriptomics/proteomics/metabolomics (pathway-level response mapping)	2–7 d	High	Very high	Offline	Very High, directly links stress to specific metabolic pathways	Low, High-dimensional, unstructured data requiring heavy post-processing	High cost; labor-intensive; offline analysis only	Deep mechanistic elucidation; Novel biomarker discovery	[67,87]
QSAR models	Prediction based on molecular structure descriptors	<1 s	Low	Low	/	Low (theoretical prediction without site-specific biological context)	High, native digital output; seamless integration into virtual models	Black-box, training dependent	High-throughput virtual screening of specific organics	[69,71,88]

## Data Availability

No new data were created or analyzed in this study. Data sharing is not applicable to this article.
